# Benefits of applying virtual reality in pelvic movement training through a Wii Fit: a randomized controlled trial

**DOI:** 10.1186/s12909-022-03109-z

**Published:** 2022-01-20

**Authors:** Hui-Ting Lin, Hsin-Jen Tsai, Yen-I Li, Wen-Pin Hu

**Affiliations:** 1 Department of Physical Therapy, I-Shou University, No. 8, Yida Road, Yan-chao District, 82445 Kaohsiung, Taiwan; 2 Department of Health Management, College of Medicine, I-Shou University, 82445 Kaohsiung, Taiwan; 3 Department of Bioinformatics and Medical Engineering, Asia University, 500, Lioufeng Road, 41354 Wufeng, Taichung, Taiwan

**Keywords:** Virtual reality, Pelvic movement training, Motor learning

## Abstract

**Background:**

Pelvic movement training has become compulsory for part of medical students. An increasing amount of research has focused on the influence of virtual reality (VR) on learning effectiveness. However, its application to pelvic floor muscles or pelvic movement training is still in its infancy. We compared the effectiveness of conventional pelvic movement training with or without VR-assisted pelvic movement training for student learning.

**Methods:**

We recruited 44 university students (16 male and 28 female participants; average age = 19.7 ± 0.31 years) who had not previously received pelvic movement education or training. The participants were randomly assigned into traditional and experimental groups to acquire pelvic movements and relevant knowledge. The traditional group received conventional classes (about 15 min), whereas the experimental group received both conventional classes and VR-assisted teaching (additional VR session took approximately 25-45 min depending on the speed of movement of each participant). The participants were asked to control the trajectory of the centre of pressure on the Wii Fit balance board and build-in games to learn pelvic movements. We conducted evaluations before, immediately after, and 2 weeks after the experiment, based on the scores of written and practical examinations. The experimental group was also asked to complete a questionnaire during the posttest.

**Results:**

We carried out two-way repeated measures ANOVA and discovered that the written examination scores indicated a significant Time × Group interaction (***p***=0.015). In each group, the written and practical examinations in the posttest and follow-up test exhibited significantly improved results compared with the baseline value (***p*** <0.001, except for traditional group of written exam in follow up test vs. baseline ***p***=0.001). The written examination in the follow-up test did not decline significantly compared with those in the posttest, but the practical examination in the follow-up test was decline significantly compared with those in the posttest (***p***=0.033). The experimental group had superior overall performance in the practical examinations than the traditional group (experimental group: mean = 76.27, 95% confidence level [CI] = 70.84–81.71; traditional group: mean = 64.21, 95% CI = 58.78–69.65). No significant difference in the written examination between two groups. The percentage for agreement ratio on the usefulness, ease of use, users’ intention to continue using the VR-assisted teaching is high (95.5-100%).

**Conclusions:**

The results of this study suggested that conventional and conventional + VR teaching were both effective. However, the incorporation of VR stimulated learning motivation and facilitated precise performance of pelvic movements. It is recommended that pelvic floor muscles training could be supplemented with VR or games to increase students’ motivation and understanding how to perform pelvic movements.

## Background

Faulty postures commonly result in anterior pelvic tilt [1]. Anterior pelvic tilt leads to lumbar lordosis and stretches abdominal muscle, causing prolonged tension in the low back extensor muscle and iliopsoas muscle. This weakens the core muscle over time, which normally induces low back pain (LBP). Researchers discovered that posterior pelvic tilt enables the coccyx to rotate backwards, which elongates the pelvic floor and activates pelvic floor muscle (PFM) [2]. Postural control muscles, such as transverse abdominis, multifidus muscle, and deep muscles of the spine, all work synchronously with PFM [3]. Therefore, the co-contraction of postural control muscles and trunk stability muscles affects PFM [4]. Training trunk stability muscles to enhance PFM is conducive to lessening urinary incontinence [5]. Furthermore, a posterior pelvic tilt has a large influence on the bioelectrical activity of PFM [4], which indicates that pelvic movements influence the contraction of PFM and the occurrence of LBP. Pelvic training can help correct pelvic posture, alleviate back pain deriving from faulty postures, and improve proprioceptive awareness and the mobility of the lumbar spine, pelvis, and hips[6].

However, comprehending and correctly performing pelvic movements is not easy. Based on our observations, some learners are incapable of performing correct movements during training even with the assistance of videos and actual demonstrations (e.g. the combined movement of the bridge position and a posterior pelvic tilt movement). VR has been incorporated into teaching in various fields to facilitate motor learning and to increase the willingness to learn [7–9]. Fortunately, the application of Wii Fit, virtual reality (VR) and electromyographic biofeedback in training can increase learner concentration and overall movement ability (coordination, sensitivity, balance, muscle strength) and/or improve patients incontinence [10, 11]. Accordingly, teachers teach students integrated current technology with motor learning theories. This may be effective in teaching students the correct techniques of PFM exercise.

The most essential aspect of motor learning is ensuring learners understand how to perform the target movements. Learners must know (through feedback) how they are performing to make adjustments accordingly. Feedback can be categorised into intrinsic and extrinsic feedback based on its form. The form of feedback applied in VR studies is mostly extrinsic. Extrinsic feedback can be subcategorised into knowledge of performance (KP) and knowledge of results (KR) [12]. Feedback can also be divided into concurrent feedback and terminal feedback according to the feedback receiving time [13]. VR can be developed by the form of KR and is usually a message about whether the action will achieve the desired goal (e.g. giving scores after participants complete an action). VR applications can also incorporate positive or negative KP about desirable/undesirable movement patterns while tasks are being performed in order to address goals [14]. To our knowledge, limited evidence was found for the application of VR to pelvic floor muscles or pelvic movement training [15–17]. Martinho et al. [15] asked participants to sit on a Wii Fit balance board and indirectly trained their PFM through abdominopelvic movements by using several Wii Fit Plus ^TM^ games. The results were significantly different from those of conventional PFM training in terms of muscular endurance. Physiofun: Pelvic Floor Training is Wii software developed in cooperation with Dr. Becker Hospital to train pelvic muscle to address postpregnancy urinary tract problems [17]. Despite the application of VR in clinical pelvic movement training, no studies have examined its effectiveness for student learning. Thus, in this study, we compared the effectiveness of conventional training and conventional training + VR training for student motor learning. We assumed that adding the VR-assisted teaching system to the conventional training is more effective than conventional training alone in improving pelvic movement learning and retaining memory of the movements of the university students.

## Methods

### Sample size

The total sample size N=44 was estimated using GPower 3.1, assuming ANOVA repeated measurements approach for between effects, within effects, and interactions. The parameters were as follows: effect size f = 0.25 (ηp^2^ = 0.058; medium effect), power = 0.8, alpha = 0.05, and correlation between repeated measures r = 0.50.

### Setting, recruitment and participants

The present study was conducted at I-Shou University. Participants were recruited from November 2019– July 2020 in lecture or network by recruitment posters. The poster provided an overview and inclusion and exclusion criteria of the study. All interested participants filled in the contact information. According to the application form, we recruited 44 university students (16 male and 28 female participants; average age = 19.7 ± 0.31 years) and excluded someone who had previously learning pelvic movement education, training, yoga or Pilates. Before the experiment, participants signed a clinical trial consent form and understood their rights. This study was approved by the Institutional Review Board of E-Da Hospital (IRB No. EMRP04108N).

### Randomize and blinded

**Fig. 1:**
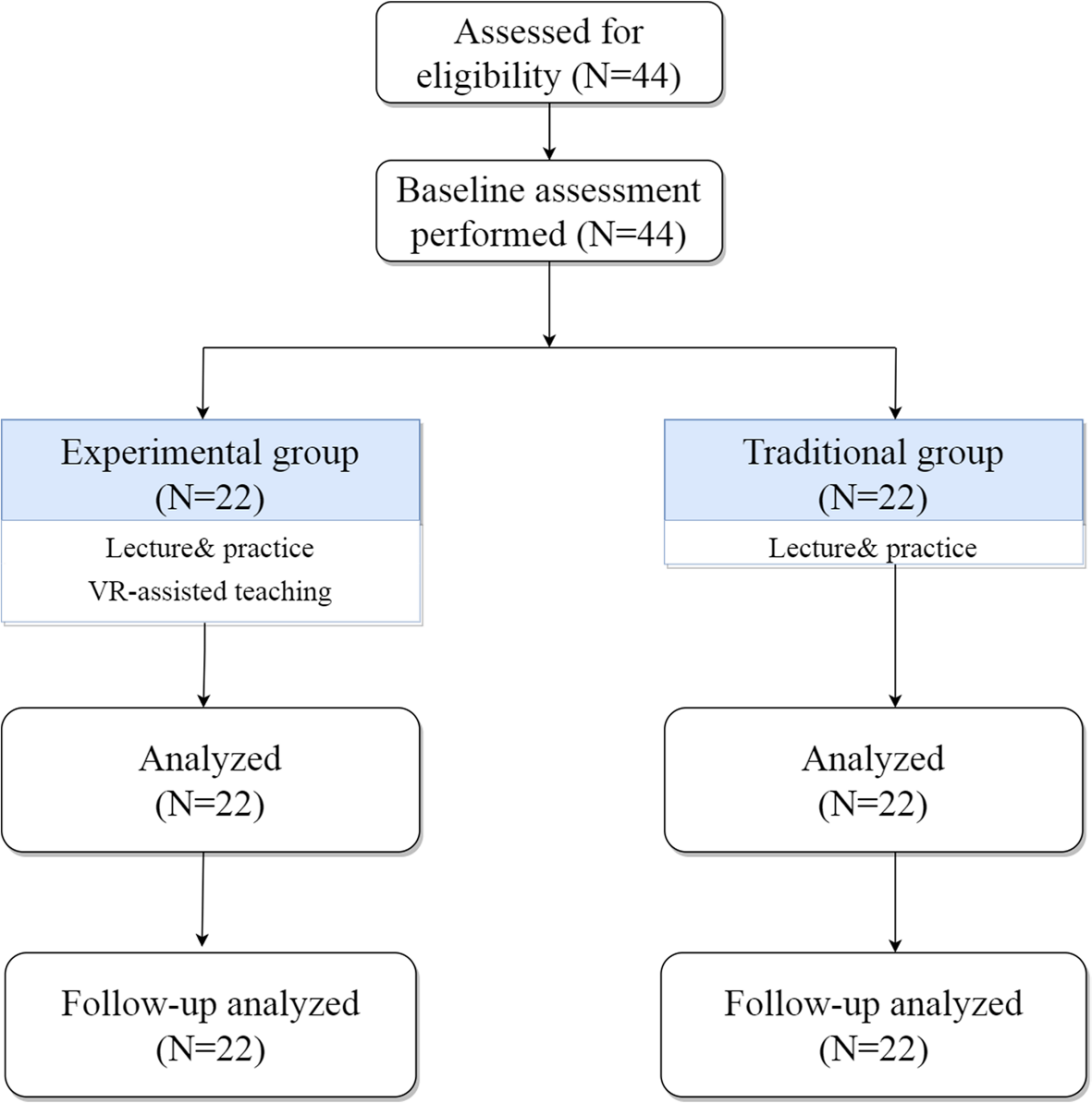
Flow chart of participants

This study was a single-blind, randomized controlled trial design and the participants were blind. We used a table of random numbers to assign equal the remaining 44 participants into experimental and traditional groups (Fig. 1).

### Intervention

All pelvic movement training and sideshows were designed based on the reference books for Taiwan National Examination in Physiotherapy. In the traditional group, the students received conventional class included lecture and practical component with text, pictures, videos and demonstrations. In the experimental group, they received the same conventional classes and added VR kits on the market (i.e. Wii Fit balance board and Wii Fit Plus ^TM^).

### Additional VR Classes

**Fig. 2:**
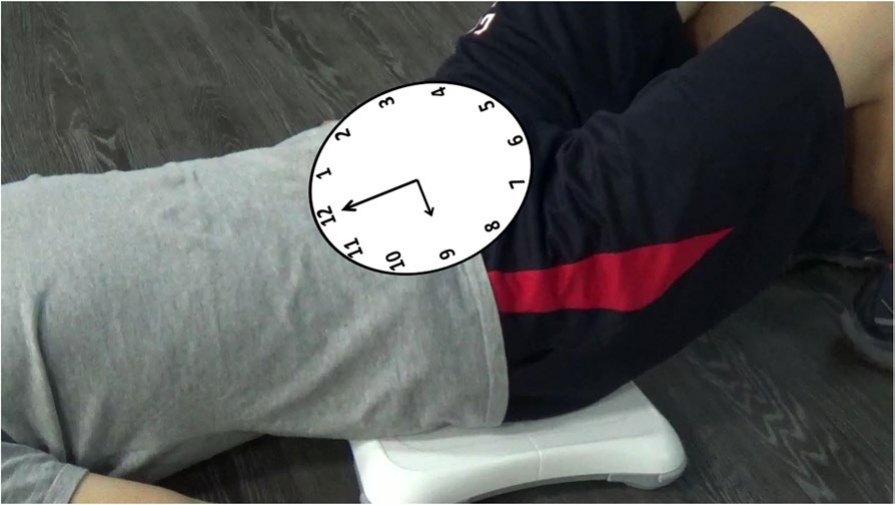
Pelvic clock exercise

**Fig. 3:**
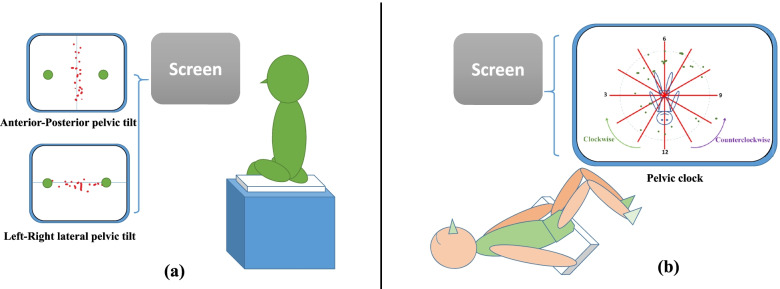
Preset trajectories of the three types of pelvic movements. **a** in sitting posture **b** in hooking lying posture

**Fig. 4:**
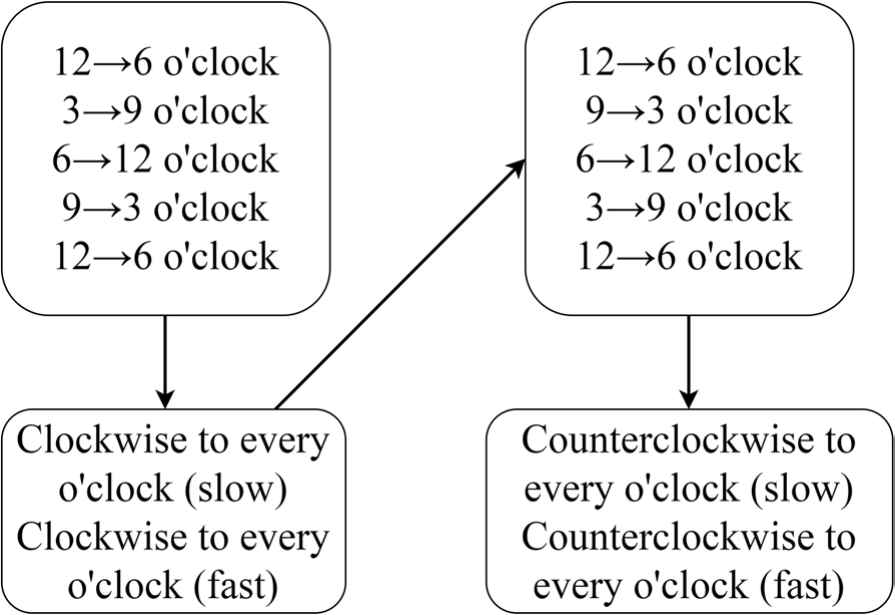
Order of the pelvic clock movements in the experimental group. Participants should repeat each set of movements three times

We employed a programming interface created using Visual C++ to capture the digital signals of the Wii Fit balance board [18]. The trajectory of the centre of pressure (COP) of the Wii Fit balance board was then exported and projected on the screen to provide participants with real-time visual feedback. Participants were given immediate visual and auditory feedback to alert them of faulty postures. They were asked to perform anterior–posterior and left–right lateral pelvic tilt movements in a sitting position and then anterior–posterior pelvic tilt, left–right pelvic rotation and pelvic clock exercise in a hook lying position on the Wii Fit balance board (Figs. 2 and 3). For more complex pelvic movements, participants were asked to practice them separately by breaking down the movements [19]. In the experiment, the pelvic clock exercise begins with a simple posterior to anterior pelvic tilt movement (12→6 o’clock), slowly progresses to a left–right rotation movement (3→9 o’clock), and ends by integrating the first two movements to perform a pelvic clock (12→3→6→9→12 o’clock). This process was from easy to difficult and from slow to fast (Fig. 4).

Before pelvic movement, COP of the balance board was set to zero. During the practices, their COP trajectories of their movements should be nearly identical to the preset trajectory shown on the projection screen. The preset trajectories of the anterior–posterior and left–right lateral pelvic tilt were respectively a vertical and a horizontal line passing through the point of origin.

**Fig. 5:**
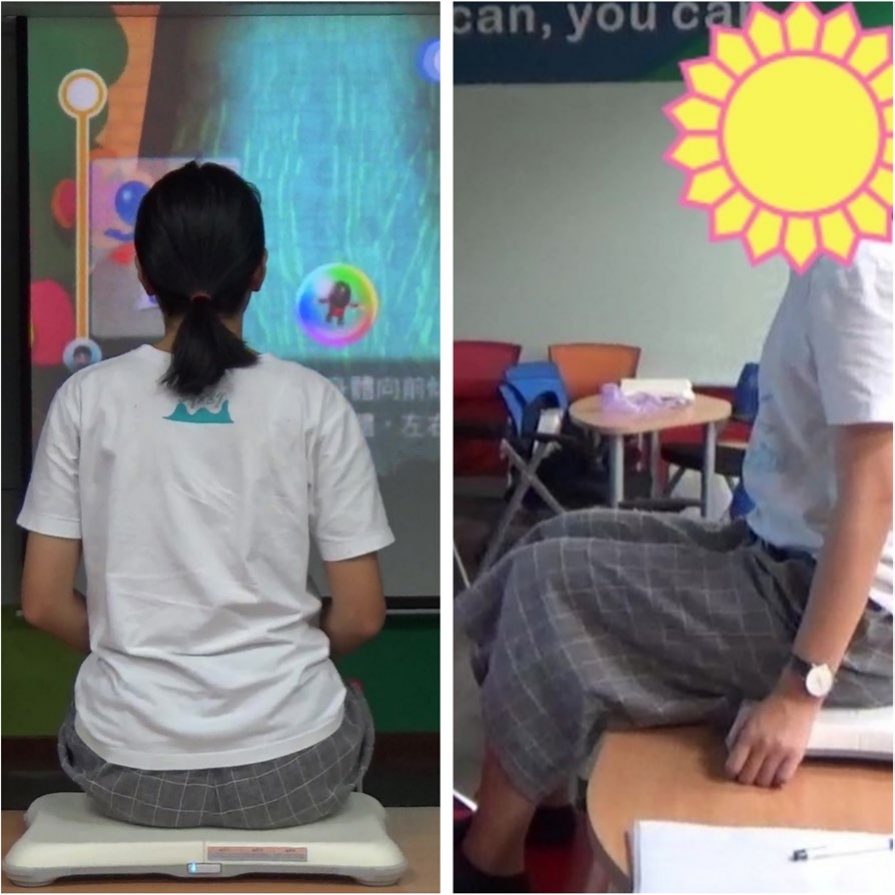
Participant in the experimental group playing Wii Fit Plus ^TM^ games

After completing the entire pelvic movement practice (Figs. 3 and 4), the participants were asked to play the built-in games in Wii Fit Plus ^TM^ (Fig. 5) (i.e. Soccer Heading ^TM^, Hula Hoop ^TM^, and Balance Bubble ^TM^) in a sitting position on the Wii Fit balance board. The three games were played in a random order with the aim of training the left–right lateral tilt, circular, and anterior–posterior pelvic movements of the participants.

### Outcome measurements

Before experiment, we asked all the participants to provide age, sex, height, weight, ischium tuberosity widths, and incidence of LBP in the preceding 3 months. We assessed the two groups before, immediately after, and 2 weeks after the experiment by using written and practical examinations. A physiotherapy professional set questions according to the class content and gave a mark in the written and practical examinations. The experimental group was then asked to complete a 5-point Likert scale questionnaire we designed with reference to those established by other researchers [20, 21]. The questionnaire was divided into three categories (usefulness, ease of use, users’ intention to continue using) with 11 questions and estimated level of agreement with the Wii Fit balance board–assisted teaching.

The written examination, a closed book examination, concerned pelvic movement training and consisted of five single-answer, multiple-choice questions with a total score of 100. The practical examination evaluated participant performance of pelvic movements though five questions, with a total score of 100; each item was evaluated by an assessor using rubrics for the accuracy of movement directions, starting position, the occurrence of compensatory movements, and the level of completion of the movements.

We evaluated the data collected from the pretest, posttest, and follow-up test to analyse the differences of the results of the written and practical examinations within group and between groups. We also confirmed whether learning was retained 2 weeks after the experiment through the results of the written and practical examinations.

### Statistical analysis

We employed IBM SPSS Statistics 22.0 to analyse the experimental data. The Shapiro-Wilk test was used to examine the normative distribution of the data. An independent sample t test was used for normally distributed data, the Mann-Whitney U-test for non-normality distributed data, and the chi-squared test for non-continuous data were used to compare the demographic data between the two groups, respectively. In addition, the scores of the written and practical examinations in pretest were examined for normality distribution. Levene’s test and Mauchly’s sphericity test were used for homogeneity between groups and time of measurement. Subsequently, two-way repeated measures ANOVA was applied to study the scores in the written and practical examinations. The factor between groups was *Group* (experimental and traditional groups), and that within groups was *Time* (pretest, posttest, and follow-up test). We also verified whether a significant *Time* × *Group* interaction existed. We then applied the least significant difference (LSD) method to *Time* (i.e. the factor within groups) for a post hoc test to inspect the differences within groups. The significance level was set at *α* = 0.05.

The agreement ratio for each question in the experimental group were calculated using formula as follows:

$$agreement\;ratio\;=\frac{number\;of\;strongly\;agree\;+\;agree\;+\;somewhat\;agree}{number\;of\;experimental\;group}$$  

## Results

### Outcome measures

All the participants completed the entire trial (16 male and 28 female participants; average age = 19.7 ± 0.31 years). Demographic characteristics between groups did not differ significantly at baseline. The scores on the written and practical examinations in the pretest were normally distributed in both groups and no significant difference existed between the groups (*p* > 0.05) (Table 1). The scores on the written and practical examinations were homogeneous, and the result did not violate the hypothesis of sphericity after being corrected by epsilon (ε; Huynh–Feldt correction).

**Table 1:** Participants’ demographic and characteristics at baseline (mean ± standard deviation)

	Experimental group (n = 22)	Traditional group (n = 22)	***p*** value
Age (year)	19.55±0.18	19.95±0.59	0.861^a^
Sex (male:female)	08:14	08:14	1.000^c^
Height (centimeter)	164.86±1.78	165±1.65	0.955^b^
Weight (kilogram)	59.61±2.23	59.3±2.83	0.541^a^
Ischium tuberosity widths (centimeter)	13.9±0.53	14.36±0.45	0.512^b^
Low back pain in the preceding 3 months (people)	5	5	1.000^c^
Baseline value of the written examination (points)	35.45±27.56	50.91±28.77	0.076^b^
Baseline value of the practical examination (points)	47.18±18.75	40.32±16.51	0.205^b^

^a^ Mann-Whitney U test. ^b^ Independent sample t test. ^c^ Chi square analysis

**Fig. 6:**
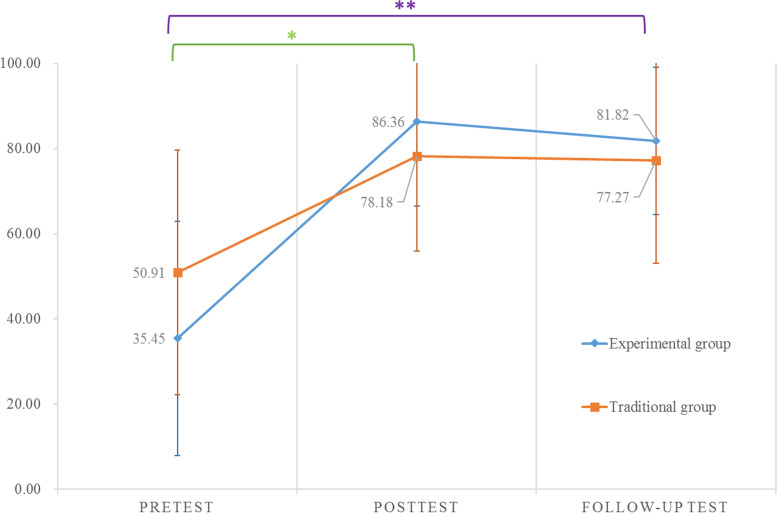
Time×Group interaction demonstrated in the written examinations (means and standard deviation bars are shown)

The tests of within-subjects effects indicated the significant influence of *Time* on the score of the written examinations (F_1.60,66.98_ = 58.19, *p* < 0.001, ηp^2^ = 0.58) and the practical examinations (F_1.84,77.28_ = 147.33, *p* < 0.001, ηp^2^ = 0.78) and that a significant *Time* × *Group* interaction existed in the written examinations (*p* = 0.015, F = 4.95; Fig. 6). However, no significant interaction was identified in the practical examinations.

In the experimental group, the written examination scores in the posttest and follow-up test exhibited significantly improved results compared with the baseline value (*p* < 0.001; *p* < 0.001). Those in the follow-up test did not decline significantly compared with those in the posttest (*p* = 0.204; Fig. 6).

The scores on the practical examinations in the posttest were significantly higher than those in the pretest, as were those in the follow-up test. However, performance in the practical examination in the follow-up test was significantly worse than that in the posttest (Table 2). In the traditional group, the written examination scores in the posttest and follow-up test were significantly improved from the baseline value (*p* < 0.001; *p* = 0.001). The written examination scores in the follow-up test exhibited no significant difference from those in the posttest (*p* = 0.825). The scores on the practical examination in the posttest were significantly higher than those in the pretest and follow-up test, and the scores on the practical examination in follow-up test were higher than those in the pretest (Table 2).

**Table 2:** Practical examination scores (points)

Variable	Category	Mean(95% Confidence Interval)	*p* value
Group	experimental group	76.27(70.84-81.71)	0.003
	traditional group	64.21(58.78-69.65)	
Time	pretest	43.75(38.37-49.13)	<0.001 ^**ab**^
	posttest	86.32(82.78-89.87)	0.033 ^**c**^
	follow-up test	80.66(74.97-86.35)	

***p*** values are presented for the differences between categories

^a^ Post hoc tests revealed significant differences between pretest and posttest on Time categories

^b^ Post hoc tests revealed significant differences between pretest and follow-up test on Time categories

^c^ Post hoc tests revealed significant differences between posttest and follow-up test on Time categories

The practical examination scores of the experimental group (mean = 76.27, 95% CI = 70.84–81.71) were significantly higher than those of the traditional group (mean: 64.21, 95% CI = 58.78–69.65; F_1,42_ = 10.02, *p* = 0.003, ηp^2^ = 0.19; Table 2). However, the written examination scores of the two groups did not significantly differ.

### Results of the questionnaire

**Fig. 7:**
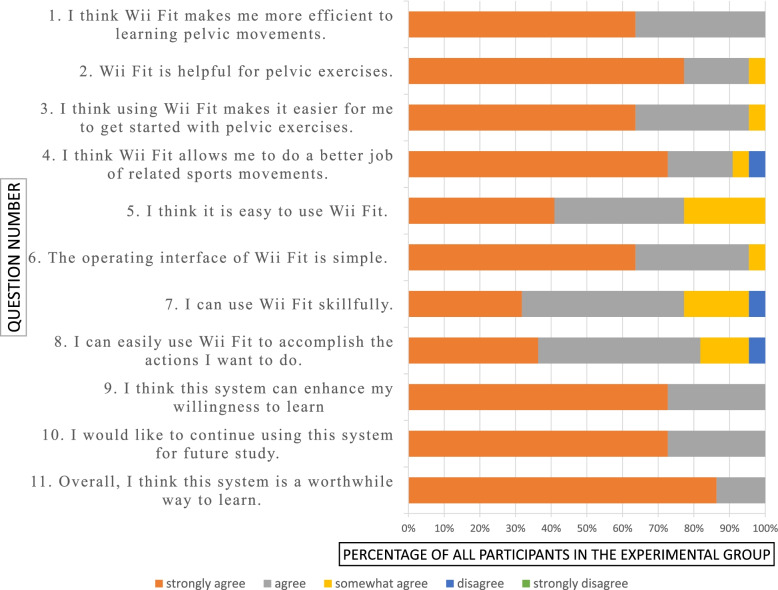
Questionnaire horizontal bar chart

The percentages for agreement ratio on the usefulness of Wii Fit were 100%, 100%, 100%, and 95.5% for question 1 to 4, respectively, which implied that most of the students considered the Wii Fit beneficial to learning pelvic movements. The percentages for agreement ratio on the ease of use of Wii Fit were 100%, 100%, 95.5%, and 95.5% for question 5 to 8, respectively, which showed most of the students felt the Wii Fit easy to use. The percentages for agreement ratio on the users’ intention to continue using were all 100% for question 9 to 11, which suggested that all the participants in the experimental group were willing to use the VR-assisted teaching system. Detailed results are demonstrated in Fig. 7. Participants in the experimental group stated, “The games are great because they make the activity easier and more interesting;” “Wii fit makes learning pelvic movements easier and increases the fun;” “Wii fit facilitates users to control their pelvic movements and directions;” “If the teaching can be clearer; learners are able to perform the movements accurately.”

## Discussion

The results revealed that both groups had significantly higher scores on the written and practical examinations in the posttest and follow-up test than those in the pretest, respectively. We consider the students who had learned the movements performed better than those who had not learnt (Fig. 6; Table 2). However, the experimental group had significantly lower scores on the practical examination in the follow-up test 2 weeks after the experiment compared with the posttest scores. In consideration that scores on both the written and practical examinations in the posttest were all significantly higher than those in the pretest (*p* < 0.001), we speculated that although VR-assisted learning generated satisfying learning outcomes in a short period of time, it reduced long-term memory retention due to more feedback the participants received [19]. Another possible reason is that the practical examinations evaluated nondeclarative body memory, which concerned in detail that was likely to be forgotten 2 weeks after the experiment [13]. We therefore assumed that nondeclarative body memory tests, such as the practical examinations, require more practice than the written examinations.

In the experimental group, VR-assisted teaching was incorporated after the conventional teaching. The experimental group had significantly higher scores on the practical examinations than the traditional group in all three tests (*p*= 0.003; Table 2). VR-assisted teaching and practice could potentially increase participants’ understanding of the movements, which would explain why the experimental group had more satisfactory performance in the practical examinations than the traditional group [19, 22]. No significant difference was observed between the two groups concerning the written examination in the posttest. The reason might be that the written examinations (multiple choice questions) could not evaluate whether participants were capable of accurately performing the movements, although they allowed quick assessments. We were unable to confirm whether correct answers were chosen out from understanding or guesswork [23]. Actual pelvic movements should be learned through practice; thus, the learning outcomes could not be estimated merely by written examinations.

Regarding the questionnaire, the experimental group expressed high approval of the VR-assisted teaching. In terms of usefulness, past study has mentioned that the Wii Fit balance board can be used to promote pelvic movement learning, and the approach successfully increased muscular endurance in PFM [15]. We applied software developed by Cooper et al. [18] to assist students in learning pelvic movements. The Wii Fit balance board, a simple and relatively cheap COP detection device available on the market, is easy to use, which exempted learners from putting too much effort into learning to operate the unfamiliar device [24]. Participants also gave high scores for the ease of use of the device. Based on these two factors, they strongly affirmed the teaching system and intention to continue using it for learning pelvic movements (Fig. 7). Furthermore, VR-assisted teaching can increase student’s intrinsic motivation. When students have high scores for interest and enjoyment, they tend to have higher scores in the course [25–27]. A study found that most nursing students at university continued to complete learning tasks to gain points even after receiving high scores because the game increased engagement and motivation [28]. Moreover, incorporating Wii Fit balance boards into learning can improve the concentration and overall movement ability of students with high-functioning autism in elementary schools. The effect remained even after the intervention ceased [10]. Hence, stimulating learning motivation benefits student learning performance. Games may be a type of method that help learners focus, and they also create a more relaxing learning environment.

The results revealed that both groups had significantly higher scores on the written and practical examinations in the posttest and follow-up test than those in the pretest, respectively. We consider the students who had learned the movements performed better than those who had not learnt (Fig. 6; Table 2). However, the experimental group had significantly lower scores on the practical examination in the follow-up test 2 weeks after the experiment compared with the posttest scores. In consideration that scores on both the written and practical examinations in the posttest were all significantly higher than those in the pretest (*p* < 0.001), we speculated that although VR-assisted learning generated satisfying learning outcomes in a short period of time, it reduced long-term memory retention due to more feedback the participants received [19]. Another possible reason is that the practical examinations evaluated nondeclarative body memory, which concerned in detail that was likely to be forgotten 2 weeks after the experiment [13]. We therefore assumed that nondeclarative body memory tests, such as the practical examinations, require more practice than the written examinations.

In the experimental group, VR-assisted teaching was incorporated after the conventional teaching. The experimental group had significantly higher scores on the practical examinations than the traditional group in all three tests (*p*= 0.003; Table 2). VR-assisted teaching and practice could potentially increase participants’ understanding of the movements, which would explain why the experimental group had more satisfactory performance in the practical examinations than the traditional group [19, 22]. No significant difference was observed between the two groups concerning the written examination in the posttest. The reason might be that the written examinations (multiple choice questions) could not evaluate whether participants were capable of accurately performing the movements, although they allowed quick assessments. We were unable to confirm whether correct answers were chosen out from understanding or guesswork [23]. Actual pelvic movements should be learned through practice; thus, the learning outcomes could not be estimated merely by written examinations.

Regarding the questionnaire, the experimental group expressed high approval of the VR-assisted teaching. In terms of usefulness, past study has mentioned that the Wii Fit balance board can be used to promote pelvic movement learning, and the approach successfully increased muscular endurance in PFM [15]. In terms of ease of use, the Wii Fit balance board, a simple and relatively cheap COP detection device available on the market exempted learners from putting too much effort into learning to operate the unfamiliar device [24]. Based on these two factors, they strongly affirmed the teaching system and intention to continue using it for learning pelvic movements (Fig. 7). Furthermore, VR-assisted teaching can increase student’s intrinsic motivation. When students have high scores for interest and enjoyment, they tend to have higher scores in the course [25–27]. A study found that most nursing students at university continued to complete learning tasks to gain points even after receiving high scores because the game increased engagement and motivation [28]. Hence, stimulating learning motivation benefits student learning performance. In the present study, VR-assisted teaching provided participants with concurrent feedback by displaying the trajectory of the COP of the Wii Fit balance board. The incorporation of games provided clear directions for application of pelvic movements. The students completed the games by using their pelvis to control the COP, and they tended to work hard to complete the tasks for higher game scores.

The common types of feedback given in VR-assisted teaching are knowledge of performance and knowledge of results, both of which are widely applied in motor learning. For example, students can adjust their movements based on images on the projection screen or the teachers remind students to move bigger during pelvic movement training [13, 29]. VR can also provide concurrent feedback that enables students to correct their movements in a timely manner with the help of an auxiliary line on the screen and combine physical guidance to make a clear standard for how movements can be completed [19]. In previous research [30], learners were asked to face an oscilloscope (kinetic feedback) and practise controlling the isometric contraction of their elbows. The group receiving concurrent feedback exhibited the highest performance during learning yet the lowest performance in tests. In another study [31], researchers discovered that for complex motor learning (e.g. beginners practising with ski simulators), the 100% concurrent high relative feedback frequency group had significantly higher scores on the retention test without feedback than did the control group; the 50% reduced feedback frequency group had no significant advantage over the control group. This indicated that high feedback frequency benefits the learning of complex movements. In the past study, applying only VR in teaching increased student motivation and interest, but the test scores of the VR group were lower than those of students in the slideshow teaching group [32]. According to the cognitive theory of multimedia learning [33] proposed by Richard Mayer, slideshow teaching can reduce cognitive load and provide learners with more efficient learning. By contrast, applying only VR to teaching may overburden the learners and distract their attention, which further reduces learning effectiveness [34]. Therefore, we incorporated VR in conventional classes to promote pelvic motor learning and compared the learning outcome with that of conventional classes only. The results revealed that incorporating VR in conventional classes increases learning motivation and has a more satisfactory learning outcome than that of conventional classes alone.

### Strengths and limitations

This study has three main strengths. First, to our knowledge, this was the first study using novelty technology accessories- virtual reality to teach pelvic motions and compared with traditional classes. Second, it was a single-blind, randomized controlled trial design which reduced possible experimental bias. Third, the questions used during the learning phase and in exams referred to highly prevalent or important clinical problems.

However, this study had a limitation. In the present study, we only compared the effectiveness of VR-assisted and conventional teaching for pelvic movement training. The results revealed that incorporating VR into conventional pelvic movement teaching has a more satisfactory outcome. Future studies are advised to add a VR teaching group without conventional classes to the experiment and explore the learning outcomes of the three groups regarding pelvic movements.

## Conclusions

The results of this study suggest that both conventional and VR-assisted teaching are significantly beneficial to learning outcomes. However, the incorporation of VR stimulates learning motivation and facilitates the learning of precise pelvic movements. Therefore, adding VR to conventional classes appears to be effective for pelvic movement learning. It is recommended that pelvic floor muscles training could be supplemented with VR or games to increase students’ motivation and understanding how to perform pelvic movements.

## Data Availability

The datasets generated and/or analysed during the current study are not publicly available due to the fact that the privacy of the individual participant cannot be compromised but are available from the corresponding author on reasonable request.

## Abbreviations

VR: virtual reality
PFM: pelvic floor muscle
LBP: low back pain
COP: centre of pressure
